# A Quantitative, Digital Method to Analyze Human Figure Drawings as a Tool to Assess Body Representations Distortions in Stroke Patients

**DOI:** 10.1109/OJEMB.2023.3277711

**Published:** 2023-05-18

**Authors:** Isabella Martinelli, Stéphanie Konik, Eleonora Guanziroli, Joseph Tharayil, Carolina Foglia, Makeda Minelik Alemu, Maria Colombo, Alessandro Specchia, Andrea Serino, Franco Molteni, Michela Bassolino

**Affiliations:** ^1^ MySpace Lab, Department of Clinical NeurosciencesUniversity Hospital Lausanne (CHUV) 30635 1011 Lausanne Switzerland; ^2^ Villa Beretta Rehabilitation CenterValduce Hospital Como 574434 22100 Costa Masnaga Italy; ^3^ Laboratory of Cognitive Neuroscience, School of Life Science, Center for Neuroprosthetics and Brain Mind InstituteSwiss Federal Institute of Technology (EPFL) 197805 1015 Geneva Switzerland; ^4^ Blue Brain ProjectÉcole polytechnique fédérale de Lausanne (EPFL) 27218 1202 Geneva Switzerland; ^5^ School of Health SciencesHES-SO Valais-Wallis 111844 1950 Sion Switzerland; ^6^ The Sense Innovation & Research Center 1011 Lausanne Switzerland

**Keywords:** Body representations, drawings, sensorimotor deficits, human figure, stroke

## Abstract

Objective: Human figure drawings are widely used in clinical practice as a qualitative indication of Body Representations (BRs) alterations in stroke patients. The objective of this study is to present and validate the use of a new app called QDraw for the quantitative analysis of drawings and to investigate whether this analysis can reveal distortions of BRs in chronic stroke patients. Results: QDraw has proven to generate reliable data as compared to manual scoring and in terms of inter-rater reliability, as shown by the high correlation coefficients. Moreover, human figure drawings from chronic stroke patients demonstrated a distortion of upper limb perception, as shown by a significantly higher arm length asymmetry compared to legs, whereas no difference was found in healthy controls. Conclusions: The present study supports the use of quantitative, digital methods (the QDraw app) to analyze human figure drawings as a tool to evaluate BRs distortions in stroke patients.

## I. Introduction

Body representations (BRs) result from patterns of neural coding from specific brain areas or from a network of brain areas, encoding and tracking the state of the body in time and space [1], [2]. These neural representations concern the perception and experience of our own body and result from a continuous flow of multisensory information that is processed bidirectionally between the body and the brain [3]. One important characteristics of BRs is their flexibility, as they are constantly updated and shaped as a function of experience (e.g., [4], [5]). Brain lesions, such as those caused by stroke, can lead to specific alterations in BRs [3].

BRs alterations in stroke patients may result in deficits of body ownership (e.g., somatoparaphrenia [6], [7], or pathological embodiment [8] or troubles in the sense of agency e.g., alien hand syndrome; [9]). Stroke patients may also report distortions in the perceptual characteristics of the body, such as its perceived dimensions, with the tendency to underestimate the perceived length of the affected arm [10], [11].

Recent studies underline the importance of assessing BRs in clinical practice, by suggesting a possible impact of BRs alterations on functional recovery after stroke [12], [13], [14], [15] but also emphasizing the need for clinically-compatible tools for BRs evaluation [16].

A practice widely used by clinicians to investigate BRs in various clinical populations (e.g., cerebral palsy [17], stroke [18]), whether in children, adolescent or adults, requires patients to simply draw a human figure (self-portrait or more generally a figure representing a human) on a sheet of paper (see Human figure test [19] for a description of the test and a review of its usage in the neuropsychological field). The traditional approach to interpret patients’ BRs distortions from the human figure drawings is mainly qualitative. However, through descriptive analyses and hierarchical classification, Morin described the human figures by proposing a categorical classification, differentiating self-portrait drawings by stroke patients and healthy subjects. The classification was based on fifteen variables, including: the position of the drawing on the paper sheet, the direction of axis of symmetry of the body (vertical or inclined toward the left or the right), the figure orientation (body mirroring or facing the viewer), the gross symmetry of the body (whether the drawing provided an impression of balance and harmony), the presence, number and aspects of body parts drawn (e.g., mouth, hand…) [18], [20]. Finally, the authors analysed the relation between drawings characteristics and clinical symptoms, so that based on this approach, several patterns of symptoms associations were identified. For instance, omissions of mouth, hands and clothes were more frequent in drawings by patients with speech disorders, while drawings by right brain damaged patients were characterized by missing body parts on one side (such as omission of one hand). Although unilateral omissions may be attributed to hemineglect, some of these patients were able to draw complete human figures of other people when asked [18], compared to incomplete self-portraits, suggesting a specific deficit in one's own body perception rather than a generic spatial bias.

Recently, this descriptive approach has been extended by the extraction of metric parameters from drawings. For example, Nuara et al., analysed self-portraits of children suffering from unilateral cerebral palsy. Their method consisted of manually measuring the inter-joint distance between the shoulder and the wrist and in computing an asymmetry index, defined as “the difference between upper limbs length expressed as a percentage of their average”. The authors found that the asymmetry index was specifically present in patients rather than in healthy controls and considered it as “a signature of hemiparetic children” [17].

Such approach could be extended by using a digital application able to measure body metric parameters of interest (such as dimensions of body parts) in a more efficient and systematic manner.

Within this context, we developed a computerized, custom-made app named QDraw (standing for Quantitative analysis of Drawings). QDraw allows to digitally analyse drawings in a standardized manner and generates numeric data for lengths and widths of body segments, that can be used with specific algorithms to compute values of interest assessing BRs.

The current study aims at presenting and validating the use of QDraw for drawings’ analysis. To reach this goal, 1) we tested the application on a sample of human figure drawings from chronic stroke patients, selected for the presence of unilateral sensorimotor deficits of the upper limb (N=56), and from healthy controls (N = 46); 2) we compared the metric parameters extracted by QDraw with those manually extracted by an expert rater, as well as the time required by each method to analyse the same subset of drawings; 3) we compared the metric parameters extracted by two independent users of QDraw app to evaluate the inter-rater reliability of the new, digital method and finally 4) we investigated whether drawings can capture distortions of BRs in chronic stroke patients, by comparing the prevalence of body features and by computing an asymmetry score of the upper and lower limbs in patients and controls.

## II. Results

*Extraction of body features and metric parameters:* For each analysed drawing, QDraw (detailed in the Materials and Methods and in Supplementary Materials sections) generated a .txt file providing target information (see Supplementary Materials, Table S.1) including whether a set of body features were visible and, when applicable, their characteristics and their metrics (e.g., whether the left or right arm was drawn, whether it was connected to the rest of the body and finally its length).

Table 1 summarizes the prevalence and statistical comparison of the main body features in controls’ and stroke patients’ drawings. A significant, higher prevalence of characteristics was found in the drawings by heathy controls rather than in those by patients regarding the general appearance of the body (hair, clothes), face (nose, mouth, eyes and ears) and the upper and lower limbs.

**TABLE 1:** Prevalence and statistical comparison of main body features extracted from drawings of healhty controls (HC; N = 46) and chronic stroke patients (P; N = 56)

Characterization of the human figure and body parts	Prevalence (%)		Statistical comparison
	HC	P	p-value
General appearance of the human figure			
Stick figure	15%	29%	1.000
Clothes	80%	50%	**0.003**
Hair	91%	50%	**<0.001**
Face characterization			
Head	100%	98%	0.649
Nose	93%	61%	**<0.001**
Mouth	100%	64%	**<0.001**
*Eyes*			
Both eyes	100%	71%	**<0.001**
Only one eye	0%	2%	0.649
No eye	0%	27%	**<0.001**
*Ears*			
Both ears	46%	14%	**0.002**
Only one ear	2%	2%	0.866
No ear	52%	84%	**0.002**
Upper body characterization			
*Arms*			
Both arms	100%	95%	0.280
Both arms connected	98%	79%	**0.005**
Only one arm	0%	4%	0.486
No arm	0%	2%	0.649
*Hands*			
Both hands	89%	63%	**0.004**
Only one hand	2%	4%	0.649
No hand	9%	34%	**0.004**
Lower body characterization			
*Legs*			
Both legs	100%	98%	0.649
Both legs connected	98%	75%	**0.002**
Only one leg	0%	0%	1.000
No leg	0%	2%	0.649
*Feet*			
Both feet	98%	66%	**<0.001**
Only one foot	0%	2%	0.649
No foot	2%	32%	**<0.001**

Significant differences on the frequency of specific elements that resist after correction are highlighted in bold font (one-tailed Fisher's exact test).

Percentages may not total 100% due to rounding in the reporting.

*Comparison between the manual and the digital method:* The average time needed for the digital evaluation with QDraw was about 3-4 min per drawing (N = 10 drawings analysed by IM: mean = 3 min 32 s; SD = 35 s), while 30 min were necessary for the manual evaluation (same 10 drawings analysed by the same user: mean = 29 min 25 s; SD = 9 min 11 s). Thus, QDraw app provided a significant advantage in terms of time required to extract the parameters from each drawing (Wilcoxon test: z = −2.803; p = 0.002).

**Figure 1.**
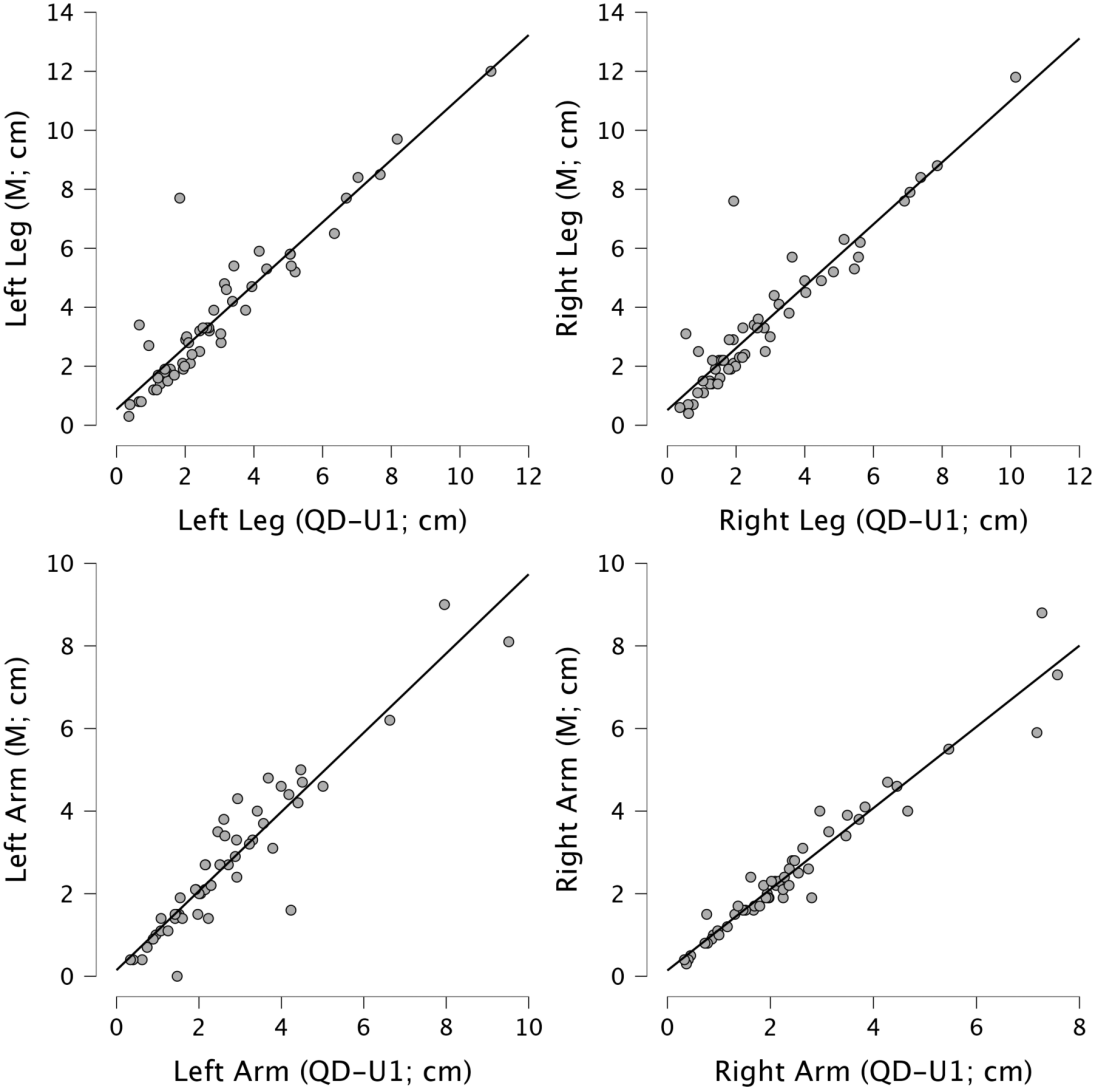
Scatterplots of correlations for leg and arm lengths (in cm) digitally extracted by user 1 (QD-U1, x-axes) and manually extracted (M, y-axes). The graphs suggest positive, linear correlations between the two sets of values.

Strong positive correlations were found between data manually extracted (M) and those digitally extracted (QD referring to QDraw; U1 referring to user 1, see Fig. 1) for the sample of stroke patients’ drawings. Spearman's correlation coefficient was higher than 0.9 for total height, head size, hips width, torso length, right leg, left arm and right arm and was higher than 0.8 for shoulders width and left leg. See Table S.2. in Supplementary Materials for detailed statistical results.

**Figure 2.**
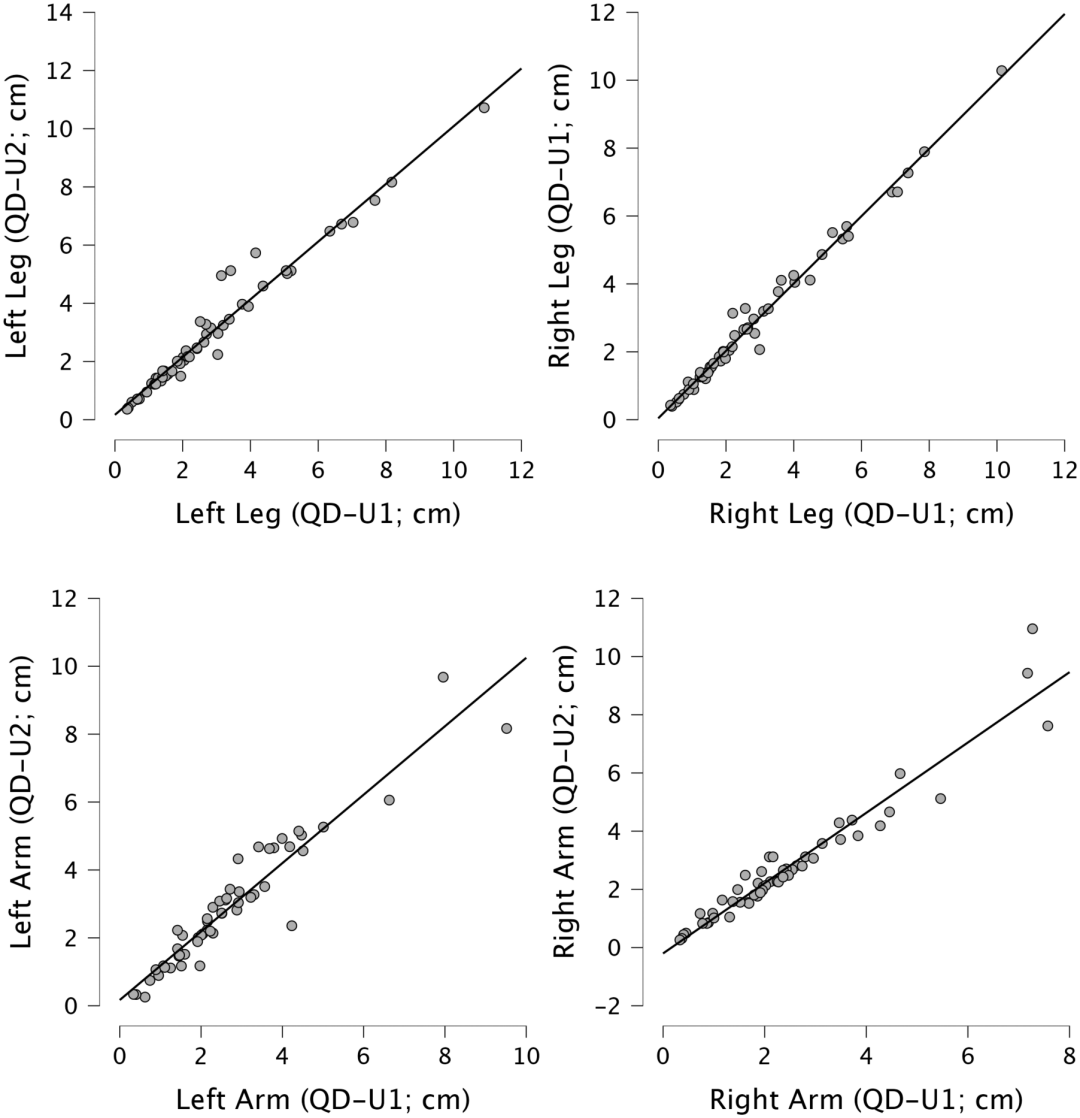
Scatterplots of correlations for legs and arms lengths (in cm) measured by user1 (QD-U1, x-axes) and user 2 (QD-U2, y-axes). The graphs show positive, linear correlations between the two sets of values.

*Comparisons between the two users of QDraw app:* Significant and strong positive correlations were found for all the metric parameters of stroke patients’ drawings extracted by the two expert users of QDraw (QD-U1, QD-U2 referring to user 2, Fig. 2). Overall, the Spearman's correlation coefficient was always higher than 0.9, except for shoulders width (rho = 0.533). See Table S.3. in Supplementary Materials for detailed statistical results.

*Distortions of BRs in chronic stroke patients captured by drawings:* Finally, to investigate whether drawings would identify an alteration of arms representation in patients suffering a unilateral sensorimotor deficit of the upper-limb, we computed and compared an Index of Asymmetry (IoA) for the upper and lower limbs (see [17] and the Materials and Methods section). We predicted the arm IoA would be significantly higher than the leg IoA, that were minimally or not affected by stroke. The Wilcoxon signed-rank test performed on arm and leg IoA showed a significant difference between the two (N= 52; z = 3.206; p = 0.001; the p-value threshold was set at 0.025 according to Bonferroni correction for two comparisons, i.e., stroke patients and healthy controls), with arm IoA significantly higher than leg IoA, meaning that upper limb asymmetry captured by drawings was greater than that of the lower limbs. This result was replicated also on the data manually measured (N= 51 z = 3.326; p = 0.001).

**Figure 3.**
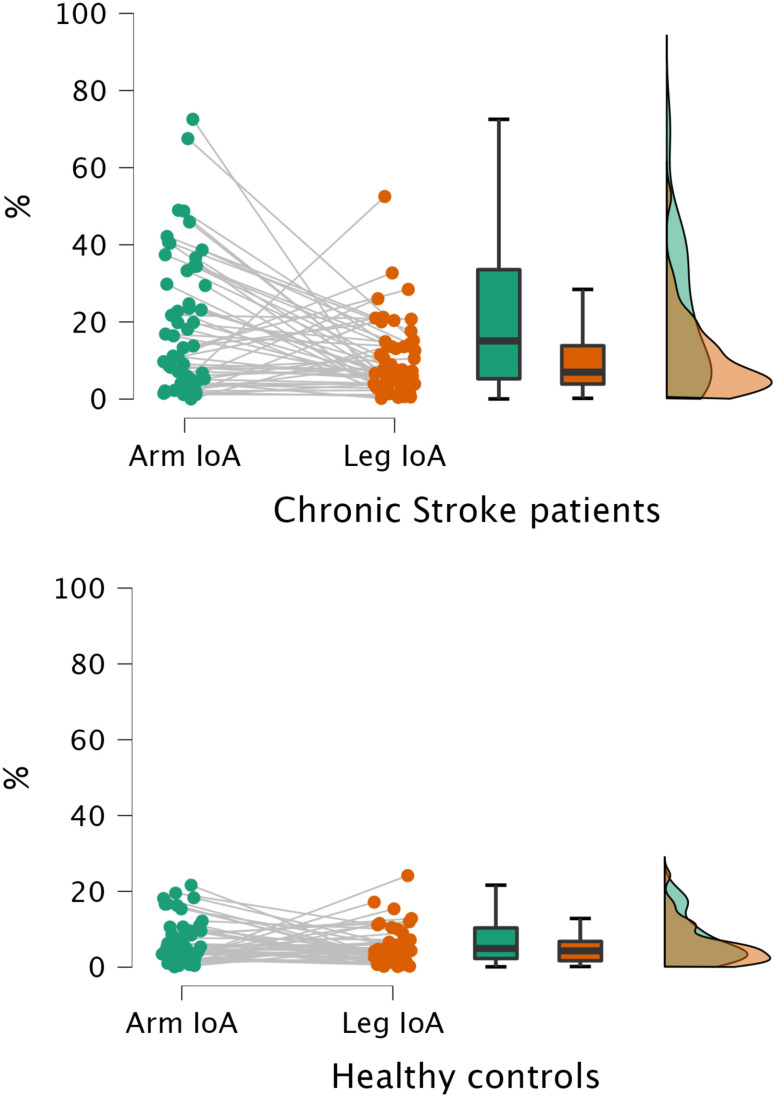
Arm (in green) and leg (in orange) IoA (IoA, in %) by chronic stroke patients and healthy controls. Each figure presents a raincloud plot, in which arm IoA cloud of points are connected to the leg IoA cloud of points, followed by boxplots showing the median (line), interquartile range (lower and upper ‘hinges’) and minimum and maximum values no further than 1.5 times the interquartile range (upper and lower whiskers) of arm and leg IoA and finally one-sided violin plots, showing the probability density of arm and leg IoA. As showed, upper limbs distortions, expressed by the arm IoA, were significantly higher than those at the lower limbs in chronic stroke patients but not in healthy subjects.

Furthermore, no significant difference between arm and leg IoA was found in healthy controls’ drawings (N= 46; z = 1.185; p = 0.241; the p-value threshold was set at 0.025 as described above). See Fig. 3 for a graphical representation of results.

## III. Discussion

Human figure drawing is a test widely used by clinicians as a qualitative indication of BRs [19]. Previous studies proposed some methods to assess BRs from drawings in stroke patients [18] and quantitatively in children suffering from unilateral cerebral palsy [17].

The aim of the current study was to develop and validate a new application (QDraw) for the quantitative analysis of human figure drawings. Our approach allows to select, through a systematic and digitalized procedure, all the visible features of the body represented (such as arms, legs and other body parts) and to extract metrics parameters as lengths and widths. These digitalized data can be then computed to calculate any index of interest.

The app has several advantages compared to the manual method, which requires to use graph paper or ruler to measure body parts dimensions.

First, it significantly reduces the time required to extract the parameters from each drawing by 25-26 min, and to analyze data that are automatically and digitally saved. Despite this huge advantage in time, QDraw provides equivalent parameters to those extracted via the manual method, as shown by the high correlation coefficients between each parameter generated manually or by a user of the app (Fig. 1). QDraw automatic coding, on the other hand, minimizes the possibility of error coming from manual rating and data reporting.

Moreover, QDraw (with its instructions, see Supplementary Materials) effectively guides users through the procedure minimizing potential variability, thus allowing to standardize the data analysis and to ensure high inter-rater reliability. Indeed, almost all the parameters extracted with QDraw by two independent users to evaluate human figure drawings by chronic stroke patients showed significant, strong, positive correlations (Fig. 2). Since a lower correlation coefficient was found for shoulders width, one possibility to further improve the app would be to insert additional information in the manual for the procedure to extract it, so to improve the inter-rater reliability obtained for this body part.

Finally, the flexibility of QDraw allows to adapt the number and type of features to be extracted based on research interests. Here, we mainly focused on asymmetry of the perceived arm length to investigate whether drawings could capture upper limb BRs distortions in chronic stroke patients suffering a persistent, severe sensorimotor deficit of the upper limb – with minimal or no motor deficits at the lower limb. Previous studies showed that these patients presented distortions of the perceived dimension of body parts, characterized by an asymmetrical underestimation of the arm length as extrapolated with the Body Landmarks Localisation task [10], [13]. Thus, here we computed an IoA for arms and legs (as control body part) [17]. We compared the upper and lower limbs indices with the hypothesis that, considering the patients’ unilateral upper limb motor deficits, distortions for the arms were greater than those for legs. This hypothesis was confirmed by QDraw-based analyses, since a significant difference was found, with arm IoA significantly higher than leg IoA. The same analyses run on human figure drawings by healthy controls showed no significant difference between arm and leg asymmetry indices. Thus, the altered representation of the upper limbs in stroke patients’ drawings was in line with what previously found in the same patients with the Body Landmarks Localization task [10]. On the other hand, no significant difference in the asymmetrical presence of other body features (eyes, ears, arms, hands, legs, feet) were noted (see Table 1), suggesting a probably preserved general symmetry of the body structure representation in stroke patients. However, overall drawings by healthy controls presented more details of the face (the nose, mouth, eyes and ears were more often represented), a more detailed representation of general appearance of the body including hair and clothes, as well as a higher presence of the upper and lower limbs than stroke patients’ drawings.

Taken together, these results suggest that reliable, quantitative measures of human figure drawings can be extracted by QDraw in a simple, systematic and efficient manner with the aim of evaluating BRs distortions following stroke. Considering the negative impact that altered BRs can have on recovery [13], [15], further studies might test if customized treatments taking into account patients’ alteration of BRs can boost the treatment outcomes.

## IV. Conclusion

QDraw represents the first attempt for the quantification and digitalization of human figure drawings analyses and the present study supports its use for detecting distortions of BRs in stroke patients. The use of the app significantly reduces the time required to measure the perceived dimensions of body parts, by also automatically storing data and allowing the implementation of ad-hoc analyses for clinical or research purposes. This would facilitate future adoption of the approach in the clinical practice. Future studies could further improve the digital method by applying advanced data extraction algorithms based on machine learning to reduce user inputs and make the procedure fully automatic.

In line with previous studies showing alterations in the metric properties of BRs following stroke [10], [11], drawings from a sample of chronic stroke patients suffering sensorimotor deficits of the upper limb revealed a distortion in upper limbs perception, as demonstrated by a significant higher arms length asymmetry compared to legs.

No arms-legs difference was observed in healthy participants (who did not present any sensorimotor impairment), thus suggesting that the difference in asymmetry index of upper and lower limbs captured in patients’ drawings may reflect the deficit in sensorimotor functions presented at the arm but not in the legs in our sample. Further studies are necessary to clarify the functional impact of this asymmetry captured by the drawings on the degree of impairment or on recovery of sensorimotor functions.

## V. Materials and Methods

Human figure drawings were collected from 56 chronic stroke patients with unilateral upper limb motor deficits who took part in the INCOGNITO study (NCT03349138, https://clinicaltrials.gov/ct2/show/NCT03349138) carried out at Villa Beretta Rehabilitation Center (Costa Masnaga, Italy), and from 46 healthy controls. A detailed description of patients’ and controls’ profile is provided in Supplementary Materials, including a summary table of patients’ demographics and clinical data (see Table S.4).

Patients (N=56, of the 60 patients reported in [10]) were provided with a sheet of paper A4 vertically and centrally presented and a pencil and were verbally asked: “Please, draw a human figure on this paper” during clinical evaluation. The same procedure was required to healthy controls (N=46). Human figure drawings were analysed using both the digital QDraw app and a manual scoring (paper graph).

QDraw was developed on Matlab (Mathworks Inc, Natick; www.mathworks.com/matlabcentral) with the purpose to provide a digital, standardized and quantitative approach to drawings’ analysis, and a detailed manual was created to guide the user through the procedure and to minimize inter-user variability (defining for each body part the points of reference to calculate the dimensions). Additionally, it presents precise rules to apply in case of exceptional features of the drawing. The app and the manual are available from the corresponding author, upon reasonable request.

The current version of QDraw allows to extract, for each analysed drawing, the presence or absence of target body features (e.g., head, hair, mouth, left and right eye and ear; left and right arm, hand, leg and foot) and when applicable their characteristic (limb connected or not to the rest of the body) and dimensions. See Table S.1. in Supplementary Materials for more detailed information.

These parameters were chosen based on previous literature [18] and according to the aims and interests of this study. The results are automatically saved in a .txt file.

**Figure 4.**
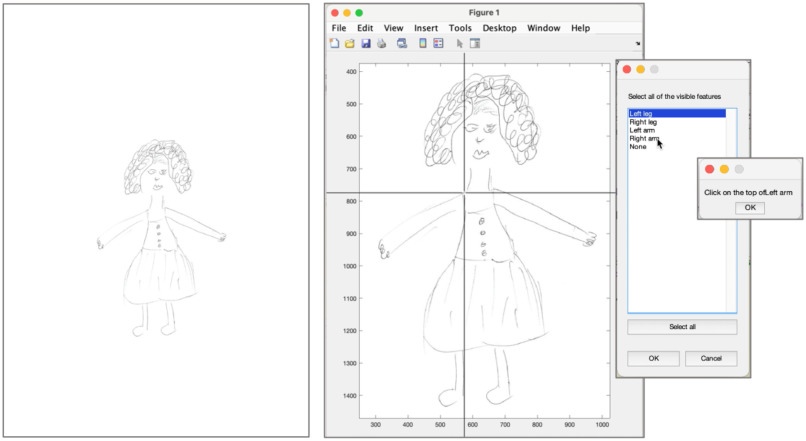
Example of a human figure drawing run on QDraw.

Fig. 4 provides an example of a human figure drawing run on QDraw. A description of how drawings’ analysis is carried out on this application and of the parameters extracted is provided in the Supplementary Materials.

To validate the use of QDraw for drawings’ analysis, the sample of human figures drawn by chronic stroke patients was run by two independent users of the app (QD-U1, IM; QD-U2, CF). QD-U1 also analysed healthy controls’ drawings with QDraw.

A third experimenter (EG) manually (M) extracted all patients’ and controls’ drawings by measuring with graph paper the same metric parameters of interest.

Statistical analyses were performed using JASP (JASP Team, 2022; https://jasp-stats.org/) and R (R Core Team, 2022, http://www.R-project.org/).

We considered the following metric parameters of interest: total height of the human figure, head size, shoulders width, hips width, torso length, left and right arms length and left and right legs length. In order to match the measurement unit of the manual method, measures from QDraw were converted from pixels to cm (considering a pixel density of 150 dpi). Additionally, we systematically assessed the time required by the digital and the manual method to analyse the same subset of drawings.

Non-parametric tests were run in case data did not follow a normal distribution (p-value of Shapiro-Wilk test > 0.05) and outliers were not removed from the sample.

To reach aim (2) and (3) described in the introduction we considered stroke patients’ drawings. First, we compared the time required to digitally and manually analyse the same subset of drawings (N=10) by the same user (IM). Non-parametric test (Wilcoxon signed-rank test) were run in case data did not follow a normal distribution (p-value of Shapiro-Wilk test > 0.05) and outliers were not removed from the sample. Secondly, we run correlation (Spearman in case of non-normally distributed data) on the data manually (M) and digitally (QD-U1) extracted, and on the data digitally extracted by the two users of the app (QD-U1, QD-U2) for the same metric parameters.

To investigate whether human figure drawings by stroke patients were less characterized than drawings by controls, we compared the frequency of each represented body part by running a one-tailed Fisher's exact test. Multiple comparisons were corrected with false discovery rates correction (FDR, see Table 1).

Finally, to reach aim (4) presented in the introduction, data by QD-U1 for arms and legs were considered. A length IoA was computed for upper and lower limbs based on the formula by Nuara et al. [17]:

\begin{equation*}
{\bm{IoA}} = \left| {\frac{{(Left - Right)}}{{(Left + Right)}}} \right| \times 2 \times 100
\end{equation*}

Data were not normally distributed. The IoA of the arms was compared to the IoA of the legs through a Wilcoxon signed-rank test. Legs were considered as a control body part since motor deficits were absent or milder at the lower limbs than at the upper limbs (see Supplementary Materials for a detailed description of patients). Similarly, to investigate upper and lower limb distortions in a non-pathological population, the same formula [17] was used to compute an arm and leg IoA for healthy controls’ drawings and the two indices were compared through a Wilcoxon signed-rank test (data were not normally distributed).

## References

[1] F. de Vignemont, “Body schema and body image-pros and cons,” Neuropsychologia, vol. 48, no. 3, pp. 669–680, Feb. 2010, doi: 10.1016/j.neuropsychologia.2009.09.022. 19786038

[2] G. Riva, “The neuroscience of body memory: From the self through the space to the others,” Cortex, vol. 104, pp. 241–260, Jul. 2018, doi: 10.1016/j.cortex.2017.07.013. 28826604

[3] M. Bassolino and A. Serino, “Representation and perception of the body in space,” in Encyclopedia of Behavioral Neuroscience, vol. 2/3, 2nd ed. New York, NY, USA: Academic, 2021, pp. 640–656, doi: 10.1016/B978-0-12-819641-0.00137-7.

[4] M. Galigani , “Effect of tool-use observation on metric body representation and peripersonal space,” Neuropsychologia, vol. 148, Nov. 2020, Art. no. 107622, doi: 10.1016/j.neuropsychologia.2020.107622. 32905815

[5] M. Martel, L. Cardinali, A. C. Roy, and A. Farnè, “Tool-use: An open window into body representation and its plasticity,” Cogn. Neuropsychol., vol. 33, no. 1/2, pp. 82–101, Feb. 2016. doi: 10.1080/02643294.2016.1167678. 27315277 PMC4975077

[6] D. Romano and A. Maravita, “The dynamic nature of the sense of ownership after brain injury. Clues from asomatognosia and somatoparaphrenia,” Neuropsychologia, vol. 132, Sep. 2019, Art. no. 107119, doi: 10.1016/j.neuropsychologia.2019.107119. 31194981

[7] R. Ronchi , “Disownership of body parts as revealed by a visual scale evaluation. An observational study,” Neuropsychologia, vol. 138, Feb. 2020, Art. no. 107337, doi: 10.1016/j.neuropsychologia.2020.107337. 31923525

[8] F. Garbarini, C. Fossataro, L. Pia, and A. Berti, “What pathological embodiment/disembodiment tell US about body representations,” Neuropsychologia, vol. 149, Dec. 2020, Art. no. 107666, doi: 10.1016/j.neuropsychologia.2020.107666. 33130159

[9] A. Hassan and K. A. Josephs, “Alien hand syndrome,” Curr. Neurol. Neurosci. Rep., vol. 16, pp. 1–10, 2016. doi: 10.1007/s11910-016-0676-z. 27315251

[10] M. Bassolino , “Body and peripersonal space representations in chronic stroke patients with upper limb motor deficits,” Brain Commun., vol. 4, no. 4, 2022, Art. no. fcac179, doi: 10.1093/braincomms/fcac179. PMC9356734 35950092

[11] G. Tosi, D. Romano, and A. Maravita, “Mirror box training in hemiplegic stroke patients affects body representation,” Front. Hum. Neurosci., vol. 11, pp. 617, Jan. 2018, doi: 10.3389/fnhum.2017.00617. 29354040 PMC5758498

[12] R. Otaki , “Relationship between body-specific attention to a paretic limb and real-world arm use in stroke patients: A longitudinal study,” Front. Syst. Neurosci., vol. 15, pp. 173, Feb. 2022, doi: 10.3389/fnsys.2021.806257. PMC8902799 35273480

[13] A. Crema , “Neuromuscular electrical stimulation restores upper limb sensory-motor functions and body representations in chronic stroke survivors,” Med, vol. 3, no. 1, pp. 58–74, Jan. 2022, doi: 10.1016/j.medj.2021.12.001. 35590144

[14] A. Farnè , “Patterns of spontaneous recovery of neglect and associated disorders in acute right brain-damaged patients,” J. Neurol., Neurosurgery Psychiatry, vol. 75, no. 10, pp. 1401–1410, Oct. 2004, doi: 10.1136/jnnp.2002.003095. PMC1738754 15377685

[15] I. Serrada, B. Hordacre, and S. Hillier, “Recovery of body awareness after stroke: An observational study,” Front. Neurol., vol. 12, Nov. 2021, Art. no. 745964, doi: 10.3389/fneur.2021.745964. PMC8666978 34912283

[16] S. Raimo , “Body representation alterations in patients with unilateral brain damage,” J. Int. Neuropsychol. Soc., vol. 28, no. 2, pp. 130–142, Feb. 2022, doi: 10.1017/S1355617721000151. 33666151

[17] A. Nuara, P. Papangelo, P. Avanzini, and M. Fabbri-Destro, “Body representation in children with unilateral cerebral palsy,” Front. Psychol., vol. 10, pp. 354, Feb. 2019, doi: 10.3389/fpsyg.2019.00354. 30837926 PMC6389686

[18] C. Morin, P. Pradat-Diehl, G. Robain, Y. Bensalah, and M. Perrigot, “Stroke hemiplegia and specular image: Lessons from self-portraits,” Int. J. Aging Hum. Develop., vol. 56, no. 1, pp. 1–41, 2003, doi: 10.2190/F0G2-GW5C-4WG0-KBWL. 12940448

[19] E. Holtz-Eakin and I. S. Baron, “Human figure drawing tests,” in Encyclopedia of Clinical Neuropsychology. Berlin, Germany: Springer, 2011, pp. 1269–1271, doi: 10.1007/978-0-387-79948-3_1552.

[20] C. Morin and Y. Bensalah, “The self-portrait in adulthood and aging,” Int. J. Aging Hum. Develop., vol. 46, no. 1, pp. 45–70, 1998, doi: 10.2190/U3P8-8YBF-0DL0-HV2P. 9534075

